# Determination of the Bioactive Compounds from *Echinacea purpurea* (L.) Moench Leaves Extracts in Correlation with the Antimicrobial Activity and the In Vitro Wound Healing Potential

**DOI:** 10.3390/molecules28155711

**Published:** 2023-07-28

**Authors:** Cristina Burlou-Nagy, Florin Bănică, Rodica Anamaria Negrean, Tünde Jurca, Laura Grațiela Vicaș, Eleonora Marian, Ildikó Bácskay, Rita Kiss, Pálma Fehér, Simona Ioana Vicaș, Florina Miere (Groza), Adriana Ramona Memete, Annamaria Pallag

**Affiliations:** 1 Doctoral School of Biomedical Sciences, University of Oradea, Universitatii Street, 1, 410087 Oradea, Romania; cristina_burlou@yahoo.com; 2 Department of Pharmacy, Faculty of Medicine and Pharmacy, University of Oradea, 29 Nicolae Jiga Street, 410028 Oradea, Romania; florinbanica1@gmail.com (F.B.); jurcatunde@yahoo.com (T.J.); laura.vicas@gmail.com (L.G.V.); marian_eleonora@yahoo.com (E.M.); 3 Department of Preclinical Disciplines, Faculty of Medicine and Pharmacy, University of Oradea, 1st December Square 10, 410068 Oradea, Romania; rodicanegrean@yahoo.com (R.A.N.); florinamiere@uoradea.ro (F.M.); 4 Department of Pharmaceutical Technology, Faculty of Pharmacy, University of Debrecen, Nagyerdei krt. 98, H-4032 Debrecen, Hungary; bacskay.ildiko@pharm.unideb.hu (I.B.); feher.palma@pharm.unideb.hu (P.F.); 5 Department of Pharmacology and Pharmacotherapy, Faculty of Medicine, University of Debrecen, Nagyerdei krt. 98, H-4032 Debrecen, Hungary; kiss.rita@med.unideb.hu; 6 Department of Food Engineering, Faculty of Environmental Protection, University of Oradea, No. 26 General Magheru Avenue, 410087 Oradea, Romania; svicas@uoradea.ro (S.I.V.); memeteadriana25@gmail.com (A.R.M.)

**Keywords:** *Echinacea purpurea* (L.) Moench, antioxidant capacity, antimicrobial activity, wound healing potential

## Abstract

This study aimed at the relationship between antioxidant capacity, antimicrobial activity, and in vitro evaluation of the wound healing effect of the extract obtained from Echinaceae purpureae folium (EPF). This study’s objective was to assess the bioactive components (total phenol and flavonoid content) and antioxidant activity of EPF extracts using the DPPH test method. The antioxidant capacity and the quantities of the compounds with antioxidant capacity were evaluated by spectrophotometric methods. Antimicrobial activity has been investigated against various pathogenic microorganisms. The minimum inhibitory concentration was determined by the microdilution method. Additionally, our work used a scratch test to examine the in vitro wound healing effects of EPF extract on NHDF cells. Statistical analysis was used to quantify the rate of migration and proliferation of fibroblast cells within the wound. Microscope pictures of fibroblast cells exposed to various EPF extract dosages were processed to estimate the width of the wound, area of the wound, and cell density inside the wound. The study proved that there was a relationship between the antioxidant, antimicrobial, and wound healing ability of EPF extracts.

## 1. Introduction

The physiologically active chemicals found in medicinal plants are abundant. Today, 11% of important medications and over 30% of pharmaceutical products are made from plants [1,2,3,4].

As members of the Asteraceae family, the species of the genus Echinacea have an important position among medicinal plants. There are nine distinct species of echinacea; however, only three are considered therapeutically effective medicinal plants: *Echinacea angustifolia DC*, *Echinacea pallida* (*Nutt.*) *Nutt*., and *Echinacea purpurea* (L.) Moench [5,6].

There are morphological and histo-anatomical distinctions between these three species, but biochemical differences stand out. The quality of the immune system response brought on by phytopharmaceutical preparations is a reflection of the biochemical variations. On dry, sandy soil and in the prairie, the members of the genus Echinacea grow naturally in the eastern United States, in Arkansas, Oklahoma, Missouri, Kansas, and Virginia.

Volume III of “Plantarum Historia Universalis Oxoniensis” (1699), written by Oxford University professor of botany R. Morison, has the first image of the plant *Echinacea* sp. “Dracunculus Virginianus latifolius petalis florum longissimis purpurascentibus” is the name of the plant in this photograph. It appears to be Rudbeckia purpurea, which Linne first described in “Species Plantarum” in 1753 and which Moench renamed *Echinacea purpurea*. From the initial name, it appears that the respective species of Echinacea was originally from Virginia. North American Indians highly valued Echinacea as a medicinal herb and used it in various insect stings, snake bites, feverish conditions, and to heal wounds. In addition to the aerial component, the roots were frequently utilized. Since there are no other Echinacea species in this area, the Native Americans of Nebraska, Missouri, and Virginia utilized *Echinacea angustifolia* while the Virginians used *Echinacea purpurea*.

In the Midwest of the United States, H.C.F. Meyer introduced the first Echinacea preparation under the name “Meyer’s Blood Purifier” in about 1870. Shortly after its “discovery” by H. C. F. Meyer it became the number one medicinal plant in popularity among physicians. Several doctors employed it because of its efficacy even though mainstream medicine refused to acknowledge its worth and it was shrouded in dispute. At the start of the 20th century, interest in this therapeutic herb shifted from America to Europe. It was initially recommended as a homeopathic treatment for lymphangitis and suppurations. The first utilization of *Echinacea angustifolia DC* was in phytotherapy. *Echinacea purpurea* (L.) Moench was first recognized as a medicinal plant after G. Madaus accidentally imported *Echinacea purpurea* (L.) Moench seed from the USA instead of *Echinacea angustifolia* seed, and the tincture made from it proved to be efficient. Along with *Ginkgo biloba*, *Allium sativum*, and *Panax ginseng*, Echinacea is currently one of the most popularly purchased medicinal herbs [7,8,9,10].

In advanced research, our team evaluated and synthesized of the data from the specialized literature, regarding the bioactive compounds, chemical composition, and pharmacological and biological properties of *Echinacea purpurea* (L.) Moench in order to highlight the possibilities and perspectives for future studies to obtain safety and pharmacologically effectiveness products [11].

From the Echinacea species, many noteworthy subgroups of bioactive substances with pharmacological properties have been identified. Alkylamides, polysaccharides, glycoproteins, flavonoids, and phenolic compounds are the four types of bioactive compounds that are now being focused on, although only *Echinacea purpurea* (L.) Moench includes all of them. Their biological characteristics have been attributed to these types of bioactive chemicals [12,13,14].

Even though *Echinacea purpurea* (L.) Moench is a rich source of bioactive compounds, only a small part of its properties has been researched. Some of these properties might be useful therapeutic tools. After reviewing the specialist literature, it was discovered that the anti-inflammatory, antioxidant, antibacterial, antiviral, antitumoral, and antiosteoporotic effects, as well as the cannabinomimetic and immunomodulating capacity, had been well-researched. According to the findings of these investigations, the active components in *Echinacea purpurea* (L.) Moench induce phagocytosis and create effective barriers against the spread of infectious and inflammatory processes [15,16,17,18,19,20,21,22,23,24]. Studies tended to place more emphasis on the consequences of internal usage than exterior use. Expanding research on dermatological effects is crucial since, in our opinion, there are solid opportunities for the creation of products based on *Echinacea purpurea* (L.) Moench that are useful in treating a variety of common dermatological conditions [25,26,27,28,29].

The aim of this study was to characterize EPF extracts in terms of bioactive compounds (total phenols and flavonoids content) and antioxidant capacity by DPPH assay method. In addition, our study investigated the antimicrobial activity of EPF extract and the wound healing effect in vitro by EPF extract in NHDF cells using a scratch assay. The rate of migration and proliferation inside the wound of fibroblast cells were quantitatively evaluated by statistical analysis. Microscope images of fibroblast cells subjected to different EP extract doses were processed in order to determine the width of the wound, the area of the wound, and the density of cells inside the wound.

## 2. Results and Discussion

### 2.1. Total Polyphenols Content

The phenolic properties are strongly related to the antioxidant activities. For this reason, the amount of total polyphenols and flavonoid substances was quantified with gallic and caffeic acid, rutin and quercetin, respectively.

Phenols are compounds that contain at least one hydroxyl group (-OH), which is conjugated to an aromatic ring group. They exist in the aerial parts of plants, such as in flowers, leaves, seeds, fruits, stems, and roots [21].

To determine the total polyphenols content (TPC) in Echinaceae purpureae folium (EPF), extracts refer to the standards of gallic acid and caffeic acid in the range 100–1000 μg/mL, the regression coefficient of 0.99671 and 0.99851, respectively, are presented in Table 1.

The values of the regression coefficients indicate good linearity between the value of the read absorbance and the concentration of the standard solutions. Using the regression equations, the TPC from the analyzed extracts was determined.

It can be seen in Table 2 that the EPF extract is a rich source of polyphenols. Thus, if it is referred to gallic acid, the TPC is 1.41 ± 0.07 mg/g dry plant material for leaf extract and if it is referred to the other standard used (caffeic acid), the TPC is 1.22 ± 0.06 mg/g dry plant material for leaves.

Approximately equal values of TPC for the two standards in the case of leaf extracts may be due to almost similar structural formulas for gallic and caffeic acid.

### 2.2. Total Flavonoids Content

For the determination of total flavonoids content (TFC), rutoside and quercetin were used as standard substances. For these, the calibration curves were drawn on the interval 100–600 μg/mL for rutoside and 5–30 μg/mL for quercetin.

Their regression coefficients together with the equations of the respective curves (Table 3) allow us to determine the TFC from the analyzed extracts (Table 4).

Reported to the routine mg/g dry plant product, EPF (1.32 ± 0.06 mg RuE/g dry plant material) was a higher total flavonoid content compared with quercetin, in which the values were 0.39 ± 0.02 mg QrE/g in dry plant material.

### 2.3. Determination of Free Radical Scavenging Activity

Antioxidant compounds found in plant materials have an important role in inhibiting and eliminating free radicals. In the present study, the antioxidant activity of ethanol extracts from EPF was investigated using the DPPH test to determine the antioxidant capacity [30]. All this proved the effectiveness of the ethanol extract from EPF extract compared with the standard reference antioxidants: ascorbic acid, gallic acid, and caffeic acid.

The antioxidant DPPH (stable free radical) is based on its ability to discolor in the presence of antioxidants. The antioxidant DPPH contains an odd electron that is responsible for absorption at 540 nm and for the deep purple color visible. When DPPH accepts an electron donated by an antioxidant compound, DPPH is discolored, which can be quantified and measured by changes in absorbance [5,12].

To determine the antioxidant capacity of the studied extracts and relate them to the three standard substances, the calibration rights corresponding to each standard substance have been recorded.

**Figure 1:**
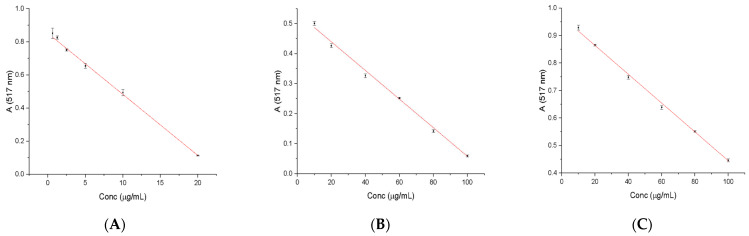
Standard calibration curves: (**A**) ascorbic acid; (**B**) gallic acid; (**C**) caffeic acid to determine antioxidant activity.

Figure 1 shows the calibration curves for ascorbic acid (0.625–20 μg/mL), gallic acid (10–100 μg/mL) and caffeic acid (10–100 μg/mL) used to determine the antioxidant capacity of the analyzed species relative to standard substances.

A good linear absorption profile of the DPPH radical diluted in methanol is observed in the range of concentrations selected for standard substances. It is desirable that the concentration of the radicals during the test vary in the accuracy range of most spectrophotometers (0.2 < A <0.8). It is known that above 0.8, the measurement is probably not accurate, and below 0.2, the differentiation is difficult to achieve. The equations of the calibration rights obtained are presented in Table 5 together with the regression coefficient.

The comparison of the antioxidant activity of the extract from leaves of the plant and the standard substances is presented.

The ethanolic extract from the EPF exhibits high antioxidant activity, presented in Table 6, compared with the three standards of AA (120.79 ± 0.0961), GA (660.71 ± 0.1182), and CA (1153.63 ± 0.14) in μg/g dry plant material. All values are expressed in ascorbic/gallic/caffeic acid equivalent.

Superior antioxidant activity related to caffeic acid is due to the polyphenols present in EPF, which is mainly derived from it. Phenolic compounds are secondary metabolites of plants. These are involved in the defense against ultraviolet radiation and pathogen attacks [30,31]. Phenolic compounds are classified in two groups: flavonoids and non-flavonoids. Flavonoids (flavones and flavonols) are generally present in plants in their glycoside form. Flavonoids and polyphenols have antioxidant activity. A large number of flavonoids are known as antioxidants, due to their strong ability to donate electrons or hydrogen atoms [32,33].

Our results show that EPF extracts are rich sourses of polyphenols and flavonoids, which also demonstrates the antioxidant activity. According to information from the literature, the results in terms of antioxidant capacity and bioactive components concentration are accurate [34,35,36]. The antioxidant capacity of EPF extract is comparable to other species of the Asteraceae family [37].

### 2.4. The Results of the HPLC Analysis

The test matrices included freeze-dried extracts obtained from the EPF as raw materials.

**Figure 2:**
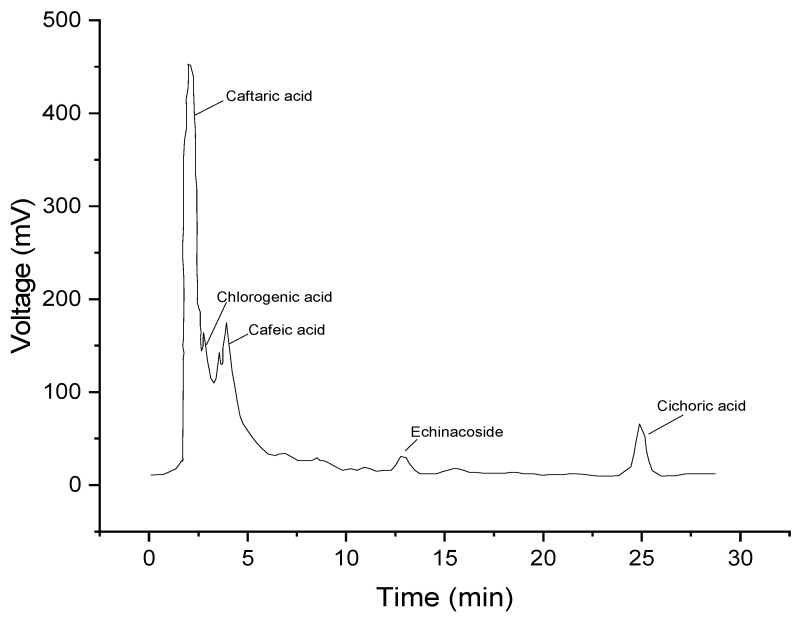
Chromatogram of the sample obtained from EPF, retention times (min).

The identification of the active compounds in the analyzed product was carried out by comparing the tr values of the recorded peaks with the individual reference standards recorded in the same chromatographic conditions, confirmed by other similar studies [38]. The predominant compounds identified are eluted as follows: caftaric acid (2064 min), chlorogenic acid (2734 min), caffeic acid (3907 min), echinacoside (12,869 min), and chicoric acid (24,943 min). The chromatogram representing the eluents of the respective compounds are shown in the Figure 2.

To determine the quantities of caffeic acid derivatives and polyphenols, the chromatograms for each reference substance were initially recorded separately, obtaining a calibration curve that was used to determine the active principles in the extract. The values obtained were caftaric acid (4.48 mg/mL), chlorogenic acid (0.08 mg/mL), caffeic acid (1.20 mg/mL), echinacoside (3.58 mg/mL), and chicoric acid (7.09 mg/mL).

HPLC fingerprinting combined with chemical pattern recognition and multicomponent content determination was a reliable, comprehensive, and the prospective method for evaluating the quality of EPF. This method provides a scientific basis for quality control and evaluation of EPF.

From the category of phenolic acids identified in the EPF extract, derivatives of caffeic acid which are caftaric acid, chlorogenic acid, caffeic acid, echinacoside, and chicoric acid), are responsible for the antioxidant activity which was highlighted in our work by DPPH assay and the determination of total phenols and flavonoids methods.

### 2.5. Evaluation of the Antimicrobial Activity

**Figure 3:**
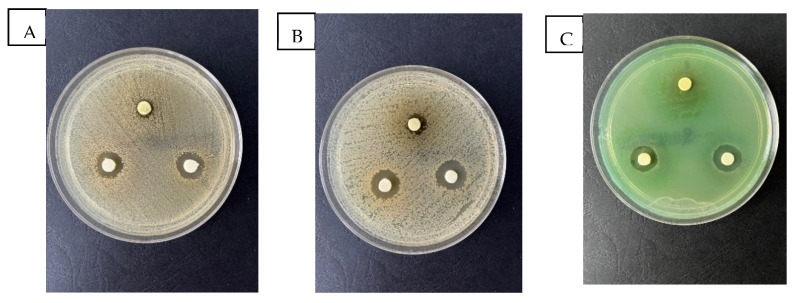
The antimicrobial activity of EPF extracts Zone of growth inhibition of (**A**) *Staphylococcus aureus*, (**B**) *Escherichia coli*, (**C**) *Pseudomonas aeruginosa*.

One of the goals of our study was to determine the antimicrobial activity of the EPF extracts. The results regarding the antimicrobial activity were evaluated by disc diffusion test, as seen in the Figure 3 and the results are summarized in Table 7.

The testing of the antibacterial activity of the EPF extract was performed on Gram-negative (*E. coli*, *P. aeruginosa*) and Gram-positive (*S. aureus*) bacteria, these are considered the main bacteria that are responsible for the production of infections in wounds [39,40].

According to the study, the therapy used for wounds must have antibacterial activity in order to asepticize the wound and influence how quickly the lesion heals [41,42]. Flavonoids, which are polyphenolic compounds, have been proven to exhibit antibacterial characteristics via a variety of mechanisms. Numerous investigations have demonstrated that flavonoids inhibit cytoplasmic membrane function, energy metabolism, and nucleic acid synthesis [43,44]. Additionally, it has been found that flavonoids inhibit the porin on cell membranes, development of biofilms, membrane permeability, and pathogenicity, all of which are vital for bacterial growth. The ability of certain flavonoids to reduce antibiotic resistance and boost the potency of available antibiotics has also been noted [45]. The creation and usage of medications based on flavonoids may offer an effective remedy for illnesses that are resistant to antibiotics.

The statistical calculation was performed with the standard deviation. 

Table 7 shows the antimicrobial activity of the EPF extract against the Gram-positive bacteria *S. aureus*, which depends on the used concentration. It can be observed that the *S. aureus* are sensitive to the EPF extract 1:4, obtaining the inhibition diameter of 15.56 ± 0.36, and EPF extract 1:8 obtaining the inhibition diameter of 15.58 ± 0.34. *S. aureus* is the most virulent staphylococcal species, which causes infections ranging from relatively minor superficial skin infections to severe conditions such as bacteremia.

Higher antimicrobial activity of the EPF extract was observed in the case of *E. coli* (Gram-negative bacteria) compared with *S. aureus*, which depends on the concentrations. The Gram-negative bacteria are sensitive to the EPF extract 1:4, obtaining the inhibition diameter of 16.09 ± 0.25, and best grown zone was at EPF 1:8 obtaining the inhibition diameter of 18.60 ± 0.38.

Thus, according to Table 7, it can be stated that the EPF extract has antimicrobial activity on Gram-positive and Gram-negative bacteria too.

Our extracts were effective against *Escherichia coli*, glucose-fermentative, and against *Pseudomonas aeruginosa* glucose-nonfermentative Gram-negative aerobes bacilli.

From the point of view of the studied EPF extracts two of them showed antimicrobial activity against all the studied microorganisms. The extracts dilusion 1:1 from leaves have lower antimicrobial activity.

The obtained zones of growth inhibition are similar to the zones obtained in other studies [46].

The antibacterial activity of the treatment applied on wounds is an important characteristic, which ensures the asepticize of the wound and determines the wound healing according to research data [47].

Our results show that the studied extracts are rich sources of substances with antioxidant activity and there are higher amounts of polyphenols and flavonoids too.

The compounds identified by HPLC analysis, derivatives of caffeic acid which are caftaric acid, echinacoside, and chicoric acid, are responsible for the antimicrobial activity of EPF.

Echinacoside is a natural phenol, isolated from Echinacea species. It is a caffeic acid glycoside from the phenylpropanoid class. Its antibiotic activity in vitro against *Staphylococcus aureus* and *Streptococcus* sp. is well-known [12,48,49].

The antimicrobial activity of the Echinacea species was also demonstrated both on Gram-negative bacteria and Gram-positive bacteria. Antimicrobial activity is provided by caffeic acid derivatives. Given the complexity of chemical profile and the strong antimicrobial effectiveness, the EPF extract can be considered a source of bioactive molecules, deserving further investigation for the versatility of application [50]. 

### 2.6. Evaluation of the Wound Healing Effect of EPF Extract Using In Vitro Scratch Assay 

In the first part of our study, the EPF extract was characterized by phytochemical composition (total phenols and flavonoids), antioxidant capacity, and antimicrobial activity. 

Based on the very good and promising results obtained, the EPF extracts in different concentrations were tested to evidence the wound healing effects. The degree of proliferation and migration of fibroblasts in vitro was tested using the “scratch” method. This test is based on an assessment of the degree and rate of fibroblasts migration under the influence of plant extracts or drugs applied in order to “heal” an artificially produced wound in vitro [51]. Thus, by performing the scratch test, we wanted to identify which concentration of EPF extract has the most intense healing and proliferative effects. 

The results of the evaluation of the wound healing effect of EPF extracts were expressed as the percentage of cell viability of the treated cells compared with the control (untreated sample), hereafter referred to as CTRL. Measurements were performed in triplicate, and the data are represented as the mean ± standard deviation (SD). The results obtained for cell viability are shown in Table 8.

**Figure 4:**
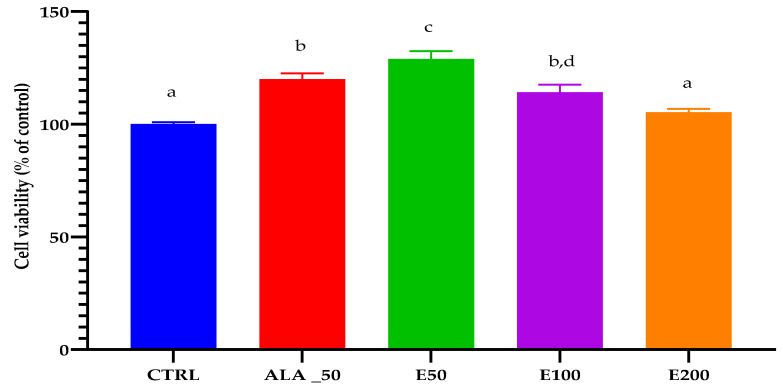
One-way ANOVA. Tukey’s multiple comparisons test. CTRL: untreated control; ALA_50: positive control allantoin 50 µg/mL; E200: Echinaceae purpureae folium extract in a concentration of 200 µg/mL; E100: Echinaceae purpureae folium extract in a concentration of 100 µg/mL; E50: Echinaceae purpureae folium extract in concentration of 50 µg/mL. Different letters represent statistical significance (*p* = 0.5).

The results for MTS are presented in Figure 4, where it can be observed that the effect of different EPF extract concentrations, ranging between 50 µg/mL and 200 µg/mL on cell viability after incubation for 24 h, was investigated (Figure 4). The percentage of cell viability did not change significantly compared with the control (CTRL0) except for the treatment with EPF at 50 µg/mL, in which a significant increase in cell viability of NHDF (normal human dermal fibroblasts) was observed.

**Figure 5:**
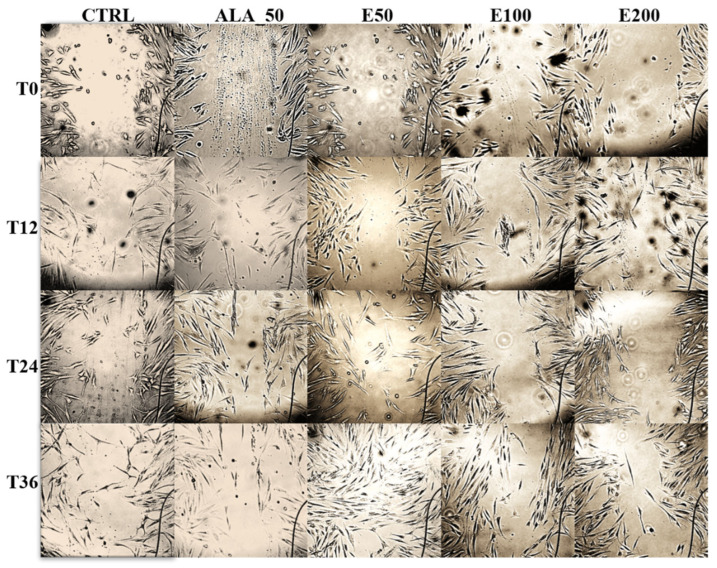
Microscopic images taken with the CytoSMART Lux3BR^®^ device for each sample applied and the evolution of wound healing in vitro as a function of time. T0: at the begining; T12: after 12 h; T24: after 24 h; T36: after 36 h; CTRL: untreated control; ALA_50: positive control allantoin 50 µg/mL; E200: Echinaceae purpureae folium extract in a concentration of 200 µg/mL; E100: Echinaceae purpureae folium extract in a concentration of 100 µg/mL; E50: Echinaceae purpureae folium extract in concentration of 50 µg/mL.

Results for the scratch method:

The images obtained depending on the treatment applied and their evolution over time are presented in Figure 5.

**Figure 6:**
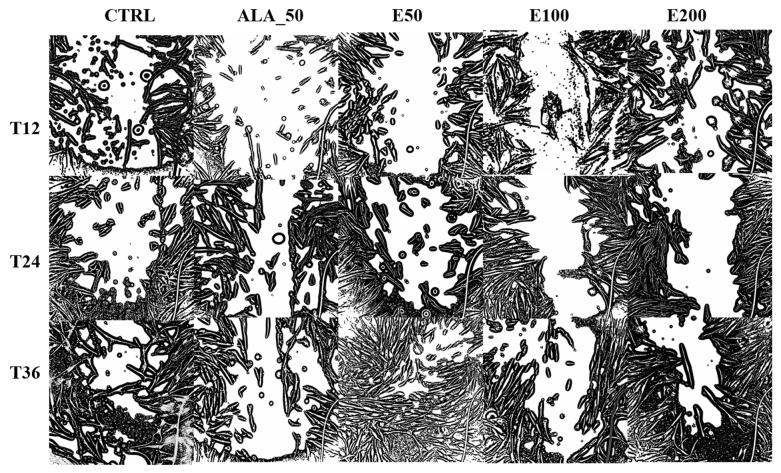
Microscopic images processed using the ImageJ 1.8 software program for each applied sample and the evolution of wound healing formed in vitro as a function of time; T12: after 12 h; T24: after 24 h; T36: after 36 h; CTRL: untreated cells; ALA_50: allantoin 50 µg/mL; E50: Echinaceae purpureae folium extract extract in a concentration of 50 µg/mL; E100: Echinaceae purpureae folium extract in a concentration of 100 µg/mL; E200: Echinaceae purpureae folium extract in a concentration of 200 µg/mL. Human dermal fibroblasts are represented in black, and the area not covered by dermal cells is represented in white.

**Figure 7:**
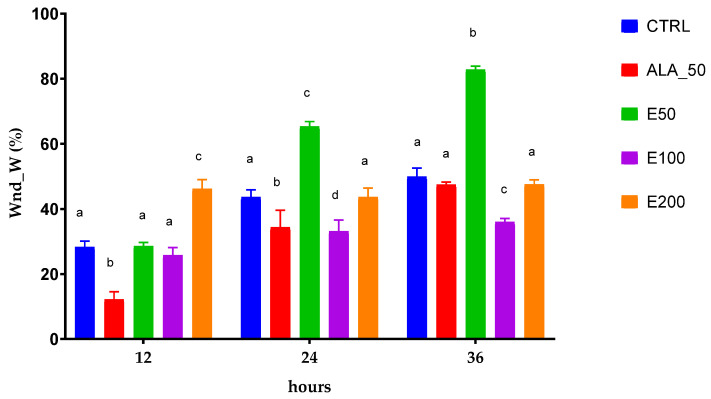
Statistically interpreted results of the wound closure rating by width (%) after 12, 24, and 36 h; CTRL: untreated cells; ALA_50: allantoin 50 µg/mL; E50: Echinaceae purpureae folium extract in a concentration of 50 µg/mL; E100: Echinaceae purpureae folium extract in a concentration of 100 µg/mL; E200: Echinaceae purpureae folium extract in a concentration of 200 µg/mL. Different letters represent statistical significance (*p* = 0.5).

**Figure 8:**
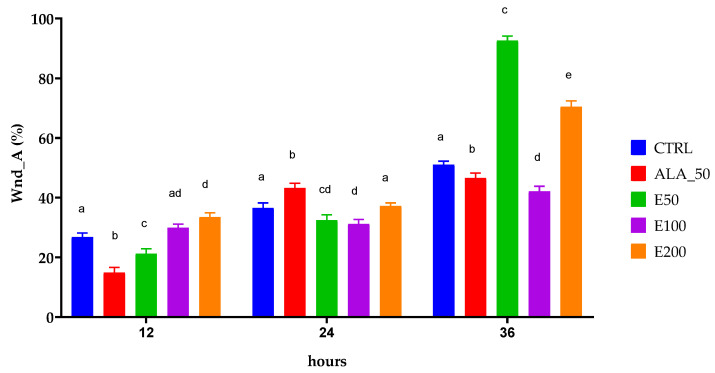
Statistically interpreted results of the wound closure by aria (%) after 12, 24, and 36 h; CTRL: untreated cells; ALA_50: allantoin 50 µg/mL; E50: Echinaceae purpureae folium extract in a concentration of 50 µg/mL; E100: Echinaceae purpureae folium extract in a concentration of 100 µg/mL; E200: Echinaceae purpureae folium extract in a concentration of 200 µg/mL. Different letters represent statistical significance (*p* = 0.5).

The images were also processed in order to calculate the width of the wound and its area (Figure 6, Table 9). Also, these images were interpreted, and the results obtained (Table 9) were statistically processed and presented in Figure 7 and Figure 8.

Based on these processed images, the closure of the wound was calculated according to the width and according to its area (%), the values being found in Table 9.

For data interpretation, the two-way ANOVA was used and Dunnett’s multiple comparisons test. Different letters represent statistical significance (*p* = 0.5). The comparison was made between samples at the same time (11, 24, and 36 h).

After applying the scratch test on NHDF and testing the degree of stimulation of the EPF extract on these cells, and after applying the image analysis and the univariate statistical analysis, it can be stated that the 50 mg/mL concentration stimulated the migration of cells into the wound and healing with a cell density inside the wound higher than the initial density in the shortest time, compared with others applied. Based on the statistical analysis, it was validated that the dose of 50 mg/mL of EPF extract healed the wound produced in vitro after 36 h at a percentage of almost 100%, namely 92.55%, compared with the other samples and induced a cell density higher than the initial one. The EPF extract has been shown to be more effective than allantoin, the positive control, a compound known to have many properties associated with wound healing [52,53]. It contains large amounts of caftaric and cichoric acid. Caffeic acid derivatives such as caftaric acid and cichoric acid with high radical scavenging activity have a well-known wound healing effect. These compounds increase collagen deposition and have a well-pronounced hyaluronidase inhibitory activity [54].

## 3. Materials and Methods

### 3.1. Plant Material

The plant material of *Echinacea purpurea* (L.) Moench used in the study comes from our own culture (Oradea, Romania). A specimen of the species was kept in the herbarium of the Faculty of Medicine and Pharmacy Oradea, Romania, registered in NYBG Steere Herbarium, UOP code 005067. Immediately after harvest, leaves (*Echinaceae purpureae folium*) (EPF) were dried separately at room temperature and ground with an electric mill with a fast-rotating knife. The extracts were obtained by maceration in ethanol. The shredded EPF (15 g) were placed in a cartridge which was left to macerate for 10 days. Extraction of the active principles was carried out with 150 mL absolute ethanol (99%). The solvent was evaporated with an R-300 rotary evaporator at a temperature of 35 °C under vacuum at 300 mBarr further than was lyophilizated. The solid residue was rerun with 100 mL of ethanol.

### 3.2. Chemicals and Equipments

In our scientific research was used: sodium acetate (Merck, Darmstadt, Germany), ascorbic acid (Fluka Chemicals, Buchs, Switzerland), caffeic acid (Sigma-Aldrich, Hamburg, Germany), gallic acid (Fluka Chemicals, Buchs, Switzerland), sodium carbonate (Reagent Bucharest, Bucharest, Romania), aluminum chloride (Merck, Darmstadt, Germany), DPPH (Cayman Chemical, Ann Arbor, MI, USA), ethanol (Chemical Company, Iasi, Romania), methanol (Chimopar, Bucharest, Romania), quercetin (Sigma-Aldrich, Hamburg, Germany), Folin–Ciocalteu reagent (Merck, Darmstadt, Germany), rutozide (Sigma-Aldrich, Hamburg, Germany), 0.9% saline solution (Merck, Darmstadt, Germany), Gram-positive-negative bacteria (Microbiologics, Saint Cloud, MN, USA), Mueller–Hinton Agar (Sanimed, Bucharest, Romania), sterile filter paper discs (HiMedia Laboratories, Thane, Maharashtra, India), sheep blood (BioMerieux, Craponne, France), fibroblast basal medium (FBM) (SC Bio Zyme SRL, Cluj-Napoca, Romania), normal human dermal fibroblasts-adult (NHDF-Ad, CC-2511, Lonza, Basel, Switzerland), recombinant human fibroblast growth factor (CC-4065) 1 mg/mL hFGF—0.5 mL (SC Bio Zyme SRL, Cluj-Napoca, Romania), Insulin (cc-4021) 5 mg/mL—0.5 mL (SC Bio Zyme SRL, Cluj-Napoca, Romania), gentamicin 50 mg/mL, Amfotericin B 50 mg/mL (CC-4081)—0.5 mL (SC Bio Zyme SRL, Cluj-Napoca, Romania), ser fetal bovine (CC-4101)—10 mL (SC Bio Zyme SRL, Cluj-Napoca, Romania), HEPES buffered saline (HEPES-BSS) (CC-5022) (SC Bio Zyme SRL, Cluj-Napoca, Romania), Trypsin/EDTA solution (T/E) (CC-5012) (SC Bio Zyme SRL, Cluj-Napoca, Romania), and trypsin neutralization solution (TNS) (CC-5002) (SC Bio Zyme SRL, Cluj-Napoca, Romania). For HPLC determinations, methanol was used for extractions and solution preparation, HPLC grade methanol for eluent preparation (Merck), glacial acetic acid (Merck), ultrapure water prepared in the laboratory, all substances are of greater than 98% purity (HPLC) from which stock solutions were prepared of: caftaric acid, chlorogenic acid, caffeic acid, chicory, and echinacoside (purchased from Sigma Aldrich).

Used equipment: UV/VIS spectrophotometer T70 + (PG Instruments Ltd., Wibtoft, UK), soft UVWIN, McFarland DEN-1 densitometer (Biosan, Riga, Latvia), rotavapor R-300 (BÜCHI Labortechnik, Flawil, Switzerland), sterile hood (Linea Blue Air Bio Activa, Lacchiarella, Italy), incubator (IncuSafe CopperAlloyStainless, Panasonic, Wiesbaden, Germany), olympus attached camera microscope XC30, apparatus for counting cells and determining their viability (EVE Automatic Cell Counter, Waltham, MA, USA), and CytoSMART LuxBR3 microscope.

For the HPLC determination, an ACME 3000 Younglin Instrument HPLC chromatographic system was used, consisting of SP 930D model, UV730D detector module. The column used in the determinations was the analytical column YMC-Pack ODS AQ 150–4.6 S—5 μm. The eluent was filtered through a Millipore system and degassed in an ultrasonic bath.

### 3.3. Determination of Total Phenols

Total phenols were determined by Folin–Ciocalteu reagent using gallic acid and caffeic acid as standards.

The stock solution of gallic acid: solutions of concentrations 0.5% gallic/caffeic acid in a mixture of ethanol:water = 1:10 (*v*/*v*) were prepared. It can be opened daily, but for storage, it can be kept in the fridge for up to two weeks.

The calibration curve was drawn for solutions of gallic/caffeic acid with concentrations of 0, 100, 200, 400, 600, 800, and 1000 μg/mL prepared from appropriate stock solutions.

From each calibration solution (gallic acid/caffeic acid), 40 µL was taken over, of which 200 µL Folin–Ciocalteu reagent and 3.16 mL of water was added, stirring vigorously. After 5 min, 600 µL solution of 20% sodium carbonate must be added. The solutions were left at 20 °C for 2 h, after that the absorbance of each at 765 nm relative to the control was determined (“0 mL” solution—does not contain gallic/caffeic acid), graphically representing the absorbance depending on the concentration [33,55].

### 3.4. Determination of Total Flavonoids

The total flavonoid content was based on the method of Chang et al., which was validated by Nuroho using routine as standard [56]. Thereby, from a stock solution of rutoside (1 mg/mL) in distilled water, 6 solutions of different concentrations were prepared: 100, 200, 300, 400, 500, and 600 μg/mL. A total of 1.5 mL of each solution mix was taken with 1.5 mL methanol, 0.1 mL 10% aluminum chloride, 0.1 mL 1M sodium acetate, and 1.25 mL distilled water. The solutions were left to rest for 60 min at room temperature and then the absorbance was read at 415 nm using the control solution consisting of the same volumes of solutions as above, except the rutoside solution which was replaced by an identical volume of methanol [57]. The total flavonoid content can be determined using quercetin as a standard as well, a stock solution of quercetin was prepared (1 mg QR/mL) in methanol, then 6 solutions were prepared of different concentrations: 30, 25, 20, 15, 10, and 5 μg/mL. From these solutions 1.5 mL was mixed with 1.5 mL of 2% AlCl_3_ solution. The mixture was left to stand for 60 min at room temperature and the absorbance was read at 420 nm [58].

### 3.5. Determination of Free Radical Scavenging Activity

The stable radical 1,1-diphenyl-2-picryl hydrazyl (DPPH), was used to determine the free radical scavenging activity of EPF extracts.

Evaluation of the antioxidant activity of the extracts from EPF was performed by the DPPH spectrophotometric method in visible light at 517 nm [8,59]. Reduction reaction is made with antioxidants. DPPH radical change is an indicator of the concentration of antioxidants needed to reduce a certain amount of radical.

To highlight the antioxidant capacity of the plant product, three standard substances were used: ascorbic acid, gallic acid, and caffeic acid. A solution was prepared of 1 mM DPPH, ascorbic acid, and 0.5 mM for gallic and caffeic acid in ethanol, which it is kept in the cold and dark for use the same day.

(a) Ascorbic acid

A stock solution was prepared of ascorbic acid, 20 μg/mL in the same solvent of which another 5 solutions of the following concentrations were prepared by successive dilutions: 10; 5; 2.5; 1.25, and 0.625 μg/mL. A total of 1 mL of ascorbic acid solution was mixed with 3 mL of 1 mM DPPH solution, and was left in the dark and at room temperature for 30 min. The absorbance of the solutions was read at 517 nm. The standard solution was ethanol, and the control solution was obtained by mixing 1 mL of ethanol with 3 mL of 1 mM DPPH solution [60].

(b) Gallic and caffeic acid

Two stock solutions were prepared, one of 100 μg/mL gallic acid and one of 100 μg/mL caffeic acid, each in ethanol from which it was prepared by successive dilutions, another 5 solutions having the following concentrations: 80, 60, 40, 20, and 10 μg/mL. A total of 50 μL gallic/caffeic acid solution was mixed with 3 mL 0.5mM DPPH solution, and left in the dark and at room temperature for 30 min. The absorbance of the solutions was read at 517 nm. The standard solution was ethanol, and the control solution was obtained by mixing 50 μL of ethanol with 3 mL of 0.5 mM DPPH solution [61].

All spectrophotometric determinations were performed in three replicates, in glass cuvettes with a 1 cm optical path.

### 3.6. HPLC Analysis

Extracts were analyzed with a HPLC ACME 3000 Younglin Instrument, consisting of SP 930D model, UV730D detector module using a YMC-Pack ODS AQ 150-4,6 S—5 μm column. The eluent was represented by methanol:water:acetic acid, 30:70:0.3. Elution conditions were isocratic with a flow rate of 1 m/min at an injected sample volume of 5 µL. Chromatograms were recorded at a wavelength of 300 nm. The linearity of the detector response was checked with the following standards: caftaric acid, chlorogenic acid, caffeic acid, chicory acid, and echinacoside.

Diluted lyophilized extract from EPF was used 1:2 (extract:methanol) [62].

### 3.7. Antimicrobial Activity

The antimicrobial activity of EPF was evaluated by the disk diffusion method using the standard methodology and by the determination of minimum inhibitory concentrations (MICs). Dilutions were made from the lyophilized extract of EPF and ethyl alcohol in a ratio of 1:1; 1:4, and 1:8.

The following test organisms were used: reference microbial strains: *Staphylococcus aureus* (+)—ATCC 25923; *Escherichia coli* (−)—ATCC 25922; and *Pseudomonas aeruginosa* (−)—ATCC 27853.

For *Staphylococcus*, *Escherichia coli*, and *Pseudomonas aeruginosa*, Mueller–Hinton Agar was used. Inoculums were prepared by direct colony suspension in salina from 20 to 24 h growth, equivalent to a 0.5 McFarland standard. Each strain was plated onto the appropriate culture media with a sterile cotton swab, and plates were dried for 10–15 min. Sterile filter paper discs of 6 mm diameter saturated with dilluated exctract of (40 μL) EPF were placed onto inoculated plates. After overnight incubation, at 37 °C, inhibition zone diameters were measured in millimeters. Filter papers impregnated with distilled water (20 μL) were used as negative controls. Each test was performed in triplicate and mean values were selected [63,64].

Minimum inhibitory concentration (MIC) determinations are defined as the lowest concentration of a drug that inhibits the visible growth of an organism after overnight incubation. Broth dilution MIC’s macrodilution was used for the following test organisms: reference microbial strain—*Staphylococcus aureus* (+)—ATCC 25923; *Escherichia coli* (−)—ATCC 25922; *Pseudomonas aeruginosa* (−)—ATCC 27853, Mueller–Hinton Broth was used. The 75 × 12 mm sterile capped tubes were used in two rows for each microbial strain to cover the range of EPF. The first tube, an inoculated tube, controlled the adequacy of the broth to support the growth of the organism, while the second tube was used to check the sterility (an uninoculated tube). 

Inoculums were prepared by direct colony suspension in salina from 20 to 24 h grown agar supplemented with 5% sheep blood, equivalent to a 0.5 McFarland standard. A total of 0.1 mL inoculum for each tube was used and all the tubes were incubated at 37 °C overnight [4,65].

### 3.8. In Vitro Evaluation of the Healing Effect of the Extract Obtained from EPF

The kit used for the cultivation of cell lines with fibroblasts (human dermal fibroblasts, normal (NHDF)) was of the type Fibroblast Growth Medium-2 BulletKit (FGM-2 BullerKit).

#### 3.8.1. Cell Culture Formation

**Figure 9:**
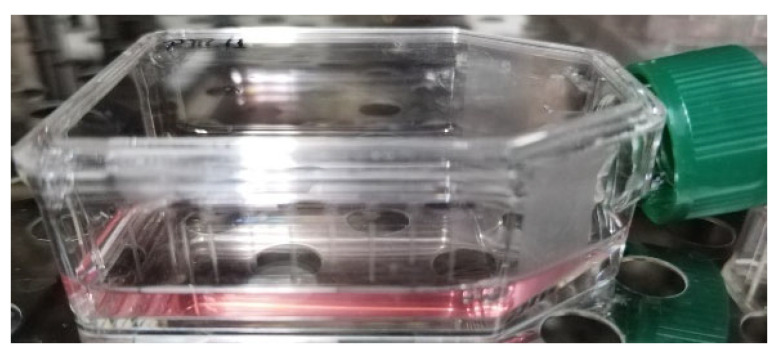
The 25 cm^2^ flask in which the cell culture was seeded.

Normal-adult human dermal fibroblasts were used for in vitro testing of the biological healing effect of EPF extract in different concentrations (E200 µg/mL, E100 µg/mL, E50 µg/mL). For cell line initiation, they were seeded in sterile 25 cm^2^ surface area flasks in a basal NHDF-type fibroblast growth medium (CC-3131, FGM, Lonza) that was enhanced with supplements (CC-4126, Lonza) according to the manufacturer’s kit (Figure 9). The cell density used was 3500 cells/cm^2^. Cell culture was maintained at 37 °C in 5% CO_2_ enriched medium in an incubator until confluence.

Once the cells reached confluence, they were used for the scratch method, viability assay, and cytotoxicity assay by the MTS method. All these steps and the entire scratch experiment were monitored using the CytoSMART Lux3BR^®^ device.

#### 3.8.2. Cell Viability Assay

**Figure 10:**
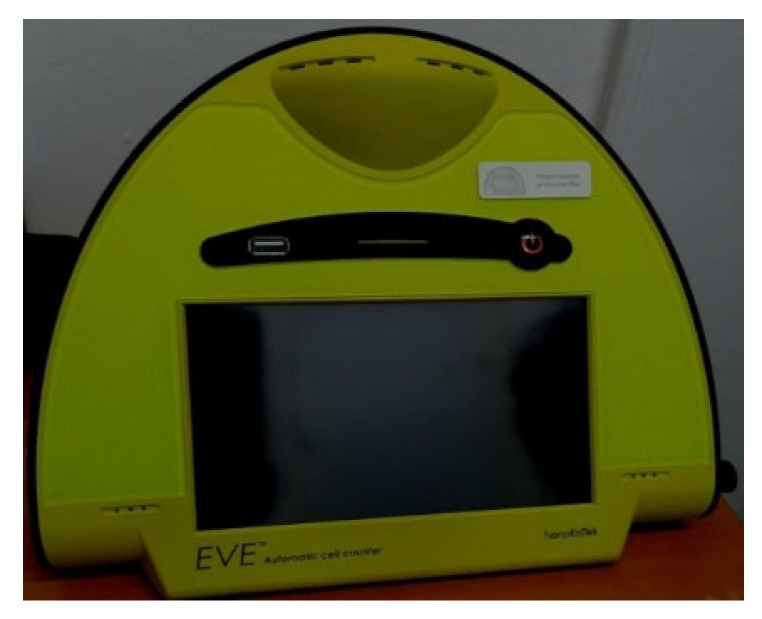
EVE Automatic Cell Counter used to measure cell viability.

Cell viability was tested using the EVE Automatic Cell Counter (NanoEnTek Inc., Seoul, Republic of Korea) (Figure 10) based on the different staining of live and dead cells when using Trypan blue dye.

This test method is an efficient and economical method of counting and testing cell viability.

To express cell viability as % viability, the calculation formula was also used:

(1) $$\mathrm{Cell}\;\mathrm{viability}\;(\%)=\frac{the\;number\;of\;living\;cells}{total\;cell\;number}\times 100$$

NHDF cells were seeded in 24-well plates at a cell density of 1 × 104 cells/well and maintained at 37 °C with 5% CO_2_ for 24 h, and then the cells were treated with EPF extract at different concentrations: E200 µg/mL, E100 µg/mL, and E50 µg/mL for 24 h. Cells were trypsinized, neutralized, and centrifuged (1000 rpm/5 min).

The obtained cell pellet was suspended in cell medium, and cell viability was determined.

#### 3.8.3. Cytotoxicity Testing of EPF Extract by the MTS Method

To perform the cytotoxicity assay, cells were seeded in sterile 98-well plates (2 × 105/mL) by adding 50 µL of suspension cell growth medium. After one day, the samples (E200 µg/mL, E100 µg/mL, E50 µg/mL, CTRL (untreated cells), and the positive control Allantoin 50 µg/mL (ALA_50)) were applied in a volume of 50 µL/ well. Cytotoxicity was determined at T 0 h and at T 24 h by the MTS method. Thus, 10 µL of a mixture of MTS (3-(4,5-dimethylthiazol-2-yl)-5-(3-carboxy-methoxyphenyl)-2-(4-sulfophenyl) 2H tetrazolium) and PMS (phenazine methosulfate) were added in a ratio of 20:1 (these being previously prepared MTS at a concentration of 2 mg/mL in Dulbecco’s phosphate buffered saline and PMS at a concentration of 0.92 mg/mL in the same solvent). After 2 h, the absorbance of the samples was measured with a microplate reader at a wavelength of 492 nm. The reference wavelength was 630 nm. Until the absorbances are read, all steps are performed sterilely and the plates are kept in the incubator at a temperature of 37 °C and 5% CO_2_ [66,67]. The results were expressed as percentage of cell viability compared with control (CTRL). Assays were performed in triplicate.

#### 3.8.4. In Vitro Testing of the Biological Healing Effect of EPF Extract Using the Scratch Method

The scratch method consists of simulating a scratch, a wound made of a single layer of confluent NHDF cells. For this, 24-well plates were seeded at a cell density of 4 × 105/cm^2^. After 48 h, the confluent cells were processed by scratching in vitro. 

Scratching was performed vertically in each well with a sterile 100 µL pipette tip. To remove detached cells, 250 µL of HEPES was used for washing. After removing the washing agent, treatments were applied in 500 µL volumes, and as an untreated control (CTRL), only cell growth medium was added in the same volume. A 50 µg/mL allantoin solution (ALA_50) was used as a positive control, and the samples were applied in concentrations of E200 µg/mL, E100 µg/mL, and E50 µg/mL. The test was performed in triplicate, and the progress of wound healing according to the applied treatment was monitored using the CytoSMART Lux3BR^®^ device at different time points: T 0 h, T 12 h, T 24 h, and T 36 h.

The following formulas were applied:

The wound closure rating by width (%) was calculated for each sample at each time point based on the formula:

(2) $$\mathrm{Pl}\_L\;(\%)=\frac{Mean\;wound\;width\;(µm)\;at\;time\;t}{Average\;wound\;width\;(µm)\;at\;time\;t=T0}\times 100$$

Also, the evaluation of wound closure according to area (%) was calculated for each sample at each time, applying the formula:

(3) $$\mathrm{Pl}\_A\;(\%)=\frac{Average\;wound\;area\;(µm²)\;at\;time\;t}{Average\;wound\;area\;(µm²)\;at\;time\;t=T0}\times 100$$

The obtained results were interpreted statistically.

### 3.9. Statistical Analysis

All measurements were performed in triplicate and the results were expressed as mean ± standard deviation, except for extract yields which were in duplicate.

The comparison of means was analyzed, and the differences were considered significant when *p* < 0.05.

## 4. Conclusions

The EPF extract was characterized in terms of bioactive compounds (total phenols and flavonoids content), and antioxidant capacity by DPPH assay with the spectrofotometric method. The HPLC analysis showed that the derivates of caffeic acid, particularly caftaric acid, echinacoside, and chicoric acid, are responsible for the antioxidant activity. These bioactive compounds are also responsible for the antimicrobial and wound healing activity of the EPF extracts. It has been proven that the relationship between the antioxidant, antimicrobial, and wound healing capacity of EPF extracts were demonstrated. We will continue the studies towards obtaining a pharmaceutical formulation for external use of EPF.

## Figures and Tables

**Table 1:** Standard substances calibration curves.

Standard Substance	Equation of the Standard Curve	Coefficients of Determination (R2)
Gallic acid	A = 0.06209 + 9.06699 × 10^−4^ × C (μg/mL)	0.99671
Caffeic acid	A = 0.13564 + 6.47403 × 10^−4^ × C (μg/mL)	0.99851

**Table 2:** TPC in mg/g dry plant material for EPF.

	Gallic Acid	Caffeic Acid
Echinaceae purpureae folium	1.41 ± 0.07 mg/g dry plant material	1.22 ± 0.06 mg/g dry plant material

**Table 3:** Standard substances calibration curves.

Standard Substance	Equation of the Standard Curve	Regression Coefficients (R^2^)
Rutin	A = 0.04786 + 0.00162 × C (μg/mL)	0.99867
Quercetin	A = −0.03537 + 0.02565 × C (μg/mL)	0.99807

**Table 4:** TFC in mg/g dry plant material for EPF.

	Rutin	Quercetin
Echinaceae purpureae folium	1.32 ± 0.06 mg RuE/g dry plant material	0.39 ± 0.02 mg QrE/g dry plant material

**Table 5:** Standard substances calibration curves.

Standard Substance	Equation of the Standard Curve	Regression Coefficients (R^2^)
Ascorbic acid	A = 0.86962 − 0.03804 × C (μg/mL)	0.99769
Gallic acid	A = 0.53474 − 0.00477 × C (μg/mL)	0.99573
Caffeic acid	A = 0.96875 − 0.00524 × C (μg/mL)	0.99871

**Table 6:** Antioxidant activity in (μg/g) from EPF.

	Ascorbic Acid	Gallic Acid	Caffeic Acid
Echinaceae purpureae folium	120.79 ± 0.0961 μg/g dry plant material	660.71 ± 0.1182 μg/g dry plant material	1153.63 ± 0.14 μg/g dry plant material

**Table 7:** The antimicrobial activity of EPF extracts; zone of growth inhibition (in mm diameter).

Extract	UM/disc	*Staphylococcus aureus* ATCC 25923	*Escherichia coli* ATCC 25922	*Pseudomonas aeruginosa* ATCC 27853
Echinaceae purpureae folium 1:1	40 μL	1.13 ± 0.21	2.16 ± 0.20	2.28 ± 0.17
Echinaceae purpureae folium 1:4	40 μL	15.56 ± 0.36	16.09 ± 0.25	14.40 ± 0.39
Echinaceae purpureae folium 1:8	40 μL	15.58 ± 0.34	18.60 ± 0.38	15.31 ± 0.17
Penicillin	10 mits	33	Not tested	Not tested
Neomicine	10 µg	17	17	Not tested
Tetracycline	30 µg	27	19	Not tested
Gentamicin	10 µg	21	21	18
Ampicillin	10 µg	16	17	Not tested
Norfloxacin	10 µg	21	29	24
Ciprofloxacin	5 µg	26	33	28
Cefadroxil	30 µg	Not tested	17	Not tested
Distilled water	-	5	5	5

**Table 8:** Cell viability obtained after treatment with EPF extract at different concentrations: CTRL-untreated control; ALA_50: positive control allantoin 50 µg/mL; E200: Echinaceae purpureae folium extract in a concentration of 200 µg/mL; E100: Echinaceae purpureae folium extract in a concentration of 100 µg/mL; E50: Echinaceae purpureae folium extract in concentration of 50 µg/mL.

Sample	% Cell Viability
CTRL	94.25 ± 7.21
ALA_50	94.687 ± 5.71
E200	93.15 ± 6.80
E100	94.55 ± 7.90
E50	95.41 ± 4.11

**Table 9:** Wound closure by width (Wnd_W) and area (%) of NHDFs after different treatments with E50, E100, E200 at T12 h, T24 h, and T36 h.

Sample	CTRL	ALA_50	E50	E100	E200
**Time (h)**	**Wound closure by width (Wnd_W)**				
**12**	28.3175 ± 1.7873	12.1901 ± 2.4587	28.5831 ± 1.1171	25.7399 ± 2.3791	46.1769 ± 2.8161
**24**	43.59547 ± 2.2742	34.3750 ± 2.3513	65.3115 ± 1.5581	33.1601 ± 3.4085	43.6698 ± 2.7696
**36**	49.89126 ± 2.6339	47.4235 ± 0.8606	82.7769 ± 1.0817	35.9606 ± 1.0599	47.4837 ± 1.4903
	**Wound closure by area or surface (Wnd_A)**				
**12**	26.7455 ± 1.4607	14.8338 ± 1.7761	21.2369 ± 1.6377	29.8948 ± 1.194	33.4771 ± 1.4980
**24**	36.5411 ± 1.6776	43.1676 ± 1.6365	32.3970 ± 1.8371	31.1618 ± 1.4967	37.160 ± 1.0570
**36**	51.06132 ± 1.1224	46.5938 ± 1.6265	92.5526 ± 1.5616	42.0614 ± 1.7360	70.4225 ± 1.9587

CTRL: untreated cells; ALA_50: allantoin 50 µg/mL; E50: Echinaceae purpureae folium extract in a concentration of 50 µg/mL; E100: Echinaceae purpureae folium extract in a concentration of 100 µg/mL; E200: Echinaceae purpureae folium extract in a concentration of 200 µg/mL.

## Data Availability

Not applicable.
